# Genome sequences of *Arthrobacter* spp. that use a modified sulfoglycolytic Embden–Meyerhof–Parnas pathway

**DOI:** 10.1007/s00203-022-02803-2

**Published:** 2022-02-24

**Authors:** Arashdeep Kaur, Phillip L. van der Peet, Janice W.-Y. Mui, Marion Herisse, Sacha Pidot, Spencer J. Williams

**Affiliations:** 1 School of Chemistry, University of Melbourne, Parkville, VIC 3010 Australia; 2 Bio21 Molecular Science and Biotechnology Institute, University of Melbourne, Parkville, VIC 3010 Australia; 3 Department of Microbiology and Immunology, University of Melbourne, Peter Doherty Institute for Infection and Immunity, Melbourne, VIC 3000 Australia

**Keywords:** Sulfur cycle, Enrichment, Nuclear magnetic resonance spectroscopy, Isotope labeling

## Abstract

Sulfoglycolysis pathways enable the breakdown of the sulfosugar sulfoquinovose and environmental recycling of its carbon and sulfur content. The prototypical sulfoglycolytic pathway is a variant of the classical Embden–Meyerhof–Parnas (EMP) pathway that results in formation of 2,3-dihydroxypropanesulfonate and was first described in gram-negative *Escherichia coli*. We used enrichment cultures to discover new sulfoglycolytic bacteria from Australian soil samples. Two gram-positive *Arthrobacter* spp. were isolated that produced sulfolactate as the metabolic end-product. Genome sequences identified a modified sulfoglycolytic EMP gene cluster, conserved across a range of other Actinobacteria, that retained the core sulfoglycolysis genes encoding metabolic enzymes but featured the replacement of the gene encoding sulfolactaldehyde (SLA) reductase with SLA dehydrogenase, and the absence of sulfoquinovosidase and sulfoquinovose mutarotase genes. Excretion of sulfolactate by these *Arthrobacter* spp. is consistent with an aerobic saprophytic lifestyle. This work broadens our knowledge of the sulfo-EMP pathway to include soil bacteria.

**Supplementary Information:**

The online version contains supplementary material available at 10.1007/s00203-022-02803-2.

## Introduction

Sulfoquinovose (SQ; 6-deoxy-6-sulfo-d-glucose) is a sulfosugar produced by photosynthetic organisms (Goddard-Borger and Williams 2017). It is primarily found as the headgroup of the sulfoglycolipid sulfoquinovosyl diacylglycerol (SQDG) in photosynthetic tissues and membranes in plants, algae and cyanobacteria (Benson et al. 1959; Goddard-Borger and Williams 2017). The annual global production of SQ is estimated at 10^10^ tons per annum (Harwood and Nicholls 1979), and thus, the degradation of this sulfosugar is an important arm of the global biogeochemical cycle. The degradation of sulfoquinovose occurs through pathways of sulfoglycolysis and provides access to its constituent carbon and generates ATP and reducing equivalents (NADH/NADPH) (Benson and Shibuya 1961; Snow et al. 2021). The sulfoglycolytic Embden–Meyerhof–Parnas (sulfo-EMP) (Denger et al. 2014; Sharma et al. 2021), Entner–Doudoroff (sulfo-ED) (Felux et al. 2015), and sulfofructose transaldolase (sulfo-SFT) (Frommeyer et al. 2020; Liu et al. 2020) pathways cleave the 6-carbon chain of SQ into two 3-carbon fragments, one of which is utilized in primary metabolism, while the other containing the sulfonate group is converted to either sulfolactate (SL) or 2,3-dihydroxypropanesulfonate (DHPS) and excreted. The sulfoglycolytic sulfofructose transketolase (sulfo-TK) pathway uses four of the six carbons in primary metabolism while the last two are excreted as isethionate (Liu et al. 2021). The sulfoglycolytic sulfoquinovose monooxygenase (sulfo-SMO) pathway results in cleavage of the C–S bond of sulfoquinovose and leads to production of glucose, and thus enables the complete breakdown of the SQ molecule (Liu et al. 2021; Sharma et al. 2022). The genes encoding these sulfoglycolytic pathways are found within clusters that typically contain genes encoding proteins for the import of SQ or its glycosides, the export of the end-products, SL or DHPS, and a specialized glycoside hydrolase termed a sulfoquinovosidase (SQase) (Speciale et al. 2016; Abayakoon et al. 2018a; Liu et al. 2021) that can cleave SQ glycosides to release SQ that can undergo sulfoglycolysis.

We report here the use of sequential enrichment culturing using minimal media containing SQ as sole carbon source to isolate new sulfoglycolytic bacteria from soil. We identify two *Arthrobacter* sp. strains, AK01 and AK04, and demonstrate that they grow on SQ and secrete SL into the growth media. We present the draft genome sequences of AK01 and AK04 that reveals that these *Arthrobacter* sp. contain a gene cluster encoding a sulfoglycolytic Embden–Meyerhof–Parnas pathway that differs from the prototypical pathway of *E. coli* through the lack of an identifiable candidate SQase, the replacement of SLA reductase with SLA dehydrogenase, and the presence of ABC transporters and TauE permeases, variations that are present within other sequenced Actinobacteria.

## Materials and methods

### Bacterial growth media

Growth media were prepared using M9 minimal salts media (2 mL), trace metal solution (0.1 mL), and vitamin solution (0.01 mL) and contained 5 mM sulfoquinovose (SQ) as sole carbon source, made up to a final volume of 10 mL with water. M9 minimal salts media contain 0.45 M Na_2_HPO_4_, 0.11 M KH_2_PO_4_, 0.09 M NH_4_Cl, 0.04 M NaCl, 0.1 M MgSO_4_, 0.1 M CaCl_2_; trace metal solution contains 0.4 mM FeCl_3_, 0.08 mM CoCl_2_, 0.08 mM CuCl_2_, 0.08 mM MnCl_2_, 0.55 mM ZnCl_2_, 0.01 mM NiCl_2_, 0.05 mM Na_2_MoO_4_ and 0.5 mM H_3_BO_3_; vitamin mixture contains 0.04 mM biotin, 0.05 mM calcium pantothenate, 0.15 mM thiamine hydrochloride, 0.36 mM *p*-aminobenzoic acid, 0.81 mM nicotinic acid, 1.49 mM pyridoxamine dihydrochloride, 0.01 B12 (cyanocobalamin).

### Isolation of *Arthrobacter* sp.

*Arthrobacter* sp. strains AK01 and AK04 were isolated from enriched culture, obtained from soil of the Botany Systems Garden (University of Melbourne).

Two soil samples were collected and approximately 1 g of soil was suspended in 5 mL of sterilized growth media containing 5 mM SQ as a sole carbon source. The culture was incubated at 30 ℃ for 4 days with agitation at 250 rpm. A subsample (100 $\mathrm{\mu L}$) was transferred into fresh vitamin-supplemented M9 media and grown for a further 4 days. This step was repeated four times and after outgrowth of the final culture for 4 days, cells were plated onto LB agar plates (10 g/L tryptone, 5 g/L NaCl, 5 g/L yeast, 15 g/L agar) and incubated overnight at 30 ℃ in dark. Single colonies were picked and inoculated into fresh vitamin-supplemented M9 media containing 5 mM SQ and incubated at 28 ℃ while shaking at 250 rpm using a Ratek orbital mixer incubator. Once the cultures were visibly turbid, cells were again plated onto LB agar, incubated overnight at 30 ℃ in dark and single colonies picked and inoculated again into fresh vitamin-supplemented M9 media containing 5 mM SQ. Once cultures were visibly turbid, frozen stocks were prepared by diluting to 10% glycerol and freezing at –80 ℃. Cell morphology was examined using scanning electron microscopy. Genomic DNA was isolated using the GenElute DNA extraction kit (Sigma) with inclusion of lysozyme and RNAase.

### Phenotypic assays

**Fig. 1:**
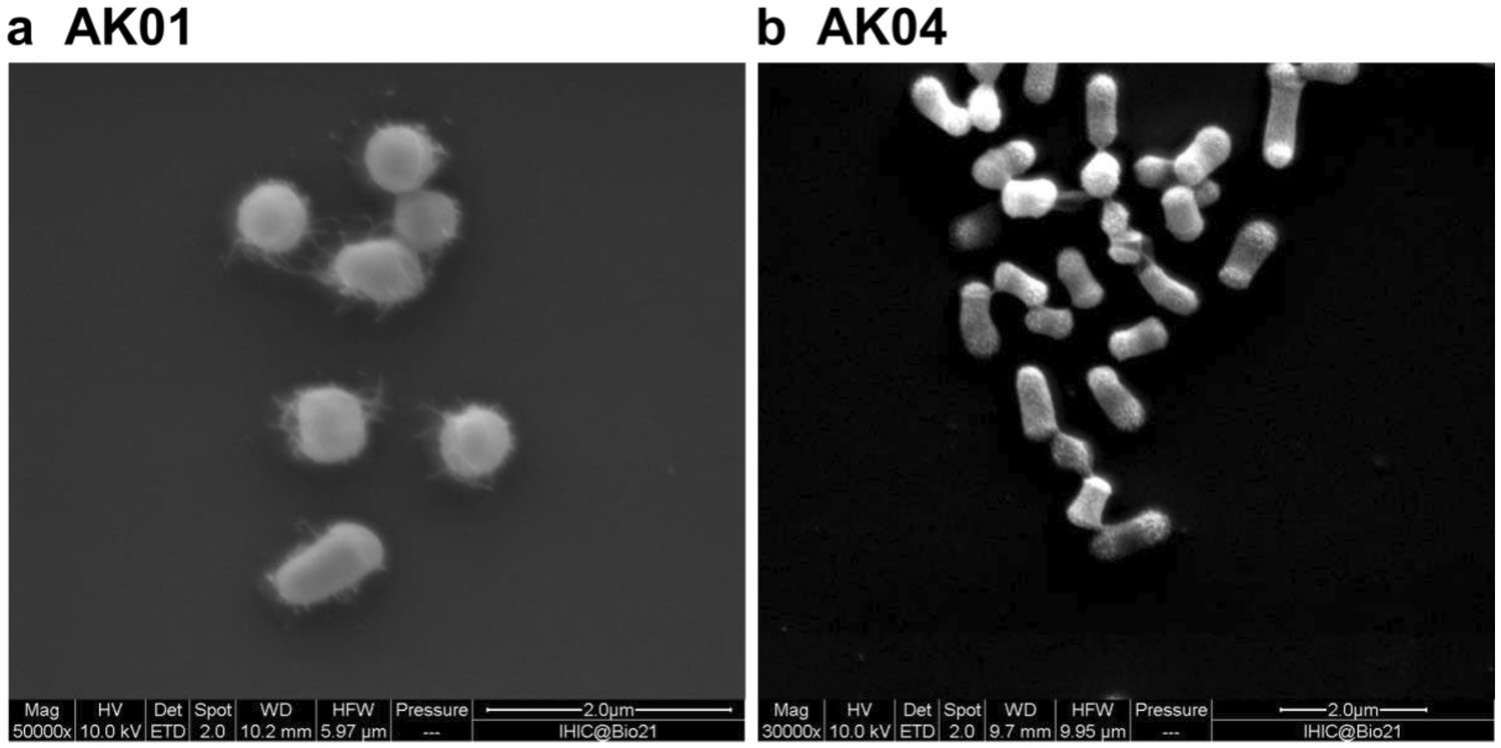
Scanning electron microscopy image of *Arthrobacter* sp. **a** strain AK01, **b** strain AK04. Cell morphology was examined using a scanning electron microscope (Quanta 200 ESEM). Cells were grown in LB media for 3 days, fixed in 0.05% glutaraldehyde in 0.1 M phosphate buffer (pH 7.4), then in 2.5% glutaraldehyde in 0.1 M phosphate buffer (pH 7.4) and allowed to react for 20 min. Fixed cells were adhered onto poly-lysine-coated slides and rinsed with water 3 times, then dehydrated by soaking in an ascending ethanol gradient (20–100%). The sample was critical point dried using a Leica CPD3000 and gold coated to thickness of 5 nm using Safematic CCU-010 compact coating unit. Images are at approximately 50,000 × magnification with scale bar shown

Bacteria were grown in vitamin-supplemented M9 minimal media containing ^13^C_6_-SQ (7.7 mM) in culture tubes at 30 ℃ for 1 day with agitation at 250 rpm. After cultures became visibly turbid, the cells were sedimented by centrifugation at 9000*g* for 10 min (using Sigma laborzentrifugen model 1-15, rotor 12124) and the supernatant was diluted with 50% D_2_O and transferred to a 5 mm NMR tube. ^13^C-NMR spectra were acquired using a 500 MHz instrument and are shown in Fig. 1a.

### Genome sequence, assembly, and annotation

Genomic DNA was sequenced using an Illumina NextSeq at the Peter Doherty Institute for Infection and Immunity, Parkville, Victoria, Australia. DNA was prepared for sequencing on the Illumina NextSeq platform using the Nextera XT DNA preparation kit (Illumina) with × 150 bp paired end chemistry and with a targeted sequencing depth of > 50 × . Draft genomes were assembled using Shovill v1.1.0 (https://github.com/tseemann/shovill) and annotated using Prokka v1.14.5 (Seemann 2014). GC percentage and ANI calculations were performed using ANI calculator (https://www.ezbiocloud.net/tools/ani) (Yoon et al. 2017). Assembled genomes have been deposited at the NCBI (GenBank accession: AK01, SAMN23041292; AK04, SAMN23041293). General features for isolated bacteria are reported in Table 1. The protein sequences of putative sulfoglycolysis proteins in AK01 and AK04 were used to search against the NCBI non-redundant database using BLASTp. Percentage identities for key sulfo proteins are given in Table S2.

**Table 1:** ^13^C-NMR (125 MHz) data of ^13^C_3_-SL produced as metabolite from ^13^C_6_-SQ

Chemical shift (*d*, ppm)	Multiplicity	Coupling constant (Hz)	Assignment
55.19	d	^1^*J*_C1,C2_ = 36.9	C3
62.42	s	NA	–
68.85	dd	^1^*J*_C2,C3_ = 52.7 ^1^*J*_C1,C2_ = 38.0	C2
72.01	s	NA	–
178.69	d	^1^*J*_C2,C3_ = 54.6	C1

Samples contain 50% D_2_O to allow frequency lock

### Discovery of related sulfoglycolytic operons

Sequences for *E. coli* sulfoquinovosidase (NP_418314.1, locus tag b3878), SQ mutarotase (NP_418315.3, locus tag b3879), SQ isomerase (NP_418316.4, locus tag b3880), SF kinase (NP_418319.2, locus tag b3883), SFP aldolase (NP_418317.1, locus tag b3881), SLA reductase (NP_418318.1, locus tag b3882) and sulfo-EMP regulator (NP_418320.2, locus tag b3884) were submitted separately as queries to the NCBI BLASTp tool. The database searched was the non-redundant protein sequence (nr) database, with *E. coli* (taxid: 562) sequences excluded. Standard algorithm parameters were used, except the maximum target sequences was set to 10,000. The results were filtered, with only protein sequences with *E*-value ≤ 5.41e-44 retained. The corresponding nucleotide accession numbers for each protein from all seven searches were extracted, and the seven lists combined and duplicates removed to give a list of candidate genome sequences. This list was converted into a MultiGeneBLAST reference library and searched using the *E. coli* sulfo-EMP gene cluster as a query. Scripts for this pipeline are available on GitHub (https://github.com/jmui-unimelb/Gene-Cluster-Search-Pipeline). Gene clusters found using this workflow were screened for clusters that contained a putative SQ isomerase, a putative SF kinase and a putative SFP aldolase, but lacked a homologous sulfoquinovosidase.

## Results and discussion

### Isolation and characterization of sulfoglycolytic bacteria

**Fig. 2:**
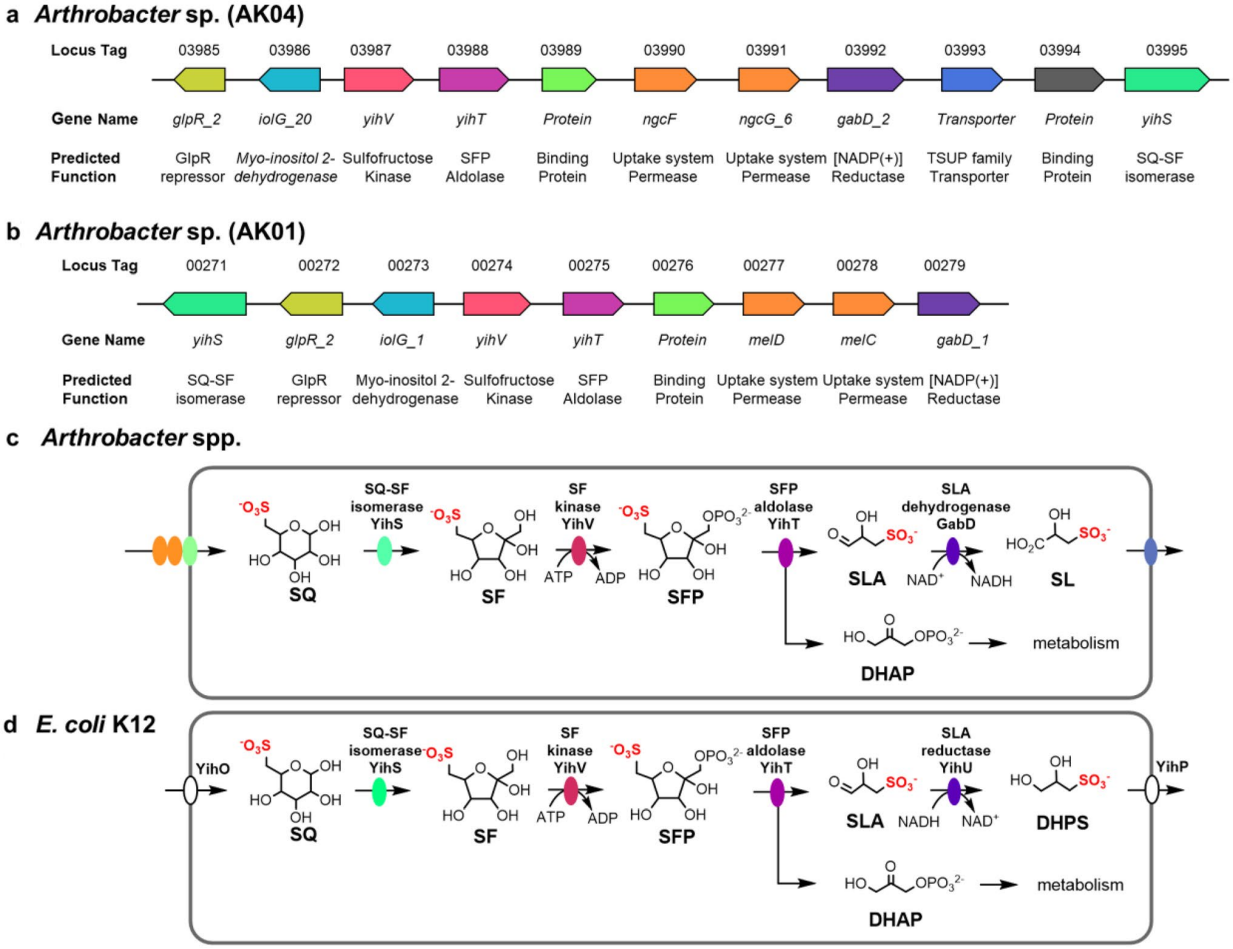
Proposed sulfoglycolytic Embden–Meyerhof–Parnas (sulfo-EMP) pathway for *Arthrobacter* spp. **a** Gene cluster encoding the sulfo-EMP pathway for *Arthrobacter* sp. AK04. **b** Gene cluster encoding the sulfo-EMP pathway for *Arthrobacter* sp. AK01. **c** Proposed sulfo-EMP pathway for *Arthrobacter* spp. **d** Comparison with EMP pathway for *E. coli* K12

Bacteria able to grow on SQ as sole carbon source were selected by using soil samples (collected from the University of Melbourne, Parkville campus) to inoculate a vitamin-enriched minimal media containing SQ as sole carbon source. Sequential subculturing into fresh SQ-minimal media, followed by plating onto LB agar and then regrowth in SQ-minimal media led to isolation of strains AK01 and AK04 that possessed a short rod-like appearance (Fig. 1). Strains AK01 and AK04 grew robustly on SQ with peak growth rates of 0.129 and 0.081 A_600_/min and achieved stationary phase after approximately 25 and 40 h, respectively (Fig. 2a, c). The absorbance at stationary phase for cultures grown on SQ were approximately half of that for cultures grown on equimolar glucose. ^13^C-NMR analysis of culture medium of AK01 and AK04 grown on ^13^C_6_-SQ (7.7 mM) media gave three signals corresponding to ^13^C_3_-SL (Fig. 2b, d, Table 1). ^13^C-NMR analysis of spent culture confirmed that substrate SQ is completely consumed by bacteria and metabolized to SL.

To investigate the genetic basis for SQ consumption by AK01 and AK04, DNA extracted from these bacteria were sequenced using the Illumina NextSeq platform. Table 2 shows the key features of the two draft genomes. On the basis of 16S rRNA gene sequence analysis the organisms were assigned as *Arthrobacter* sp. The 16S rRNA genes of the two strains were 98.8% identical over 1520 bp, which suggests that these organisms are of the same species (Kim et al. 2014). However, average nucleotide identity (ANI) between the two organisms is only 76.5%, conflicting with the 16S rDNA gene results and suggesting that these two organisms differ sufficiently to be considered separate species (Jain et al. 2018).

**Table 2:** Key features of the AK01 (GenBank accession: SAMN23041292) and AK04 (GenBank accession: AK01, SAMN23041293) draft genome assemblies

	AK01	AK04
Genome size	5,105,913	4,700,363
Number of contigs	169	130
GC %	62.9	65.5
Number of ORFs	4734	4318
Number of putative genes	4669	4261
Number of putative tRNA	59	53
Number of putative rRNA	5	3
Number of putative tmRNA	1	1
Number of genes assigned a function (%)	2478 (53%)	2219 (52%)

### Genomic features related to SQ metabolism

**Fig. 3:**
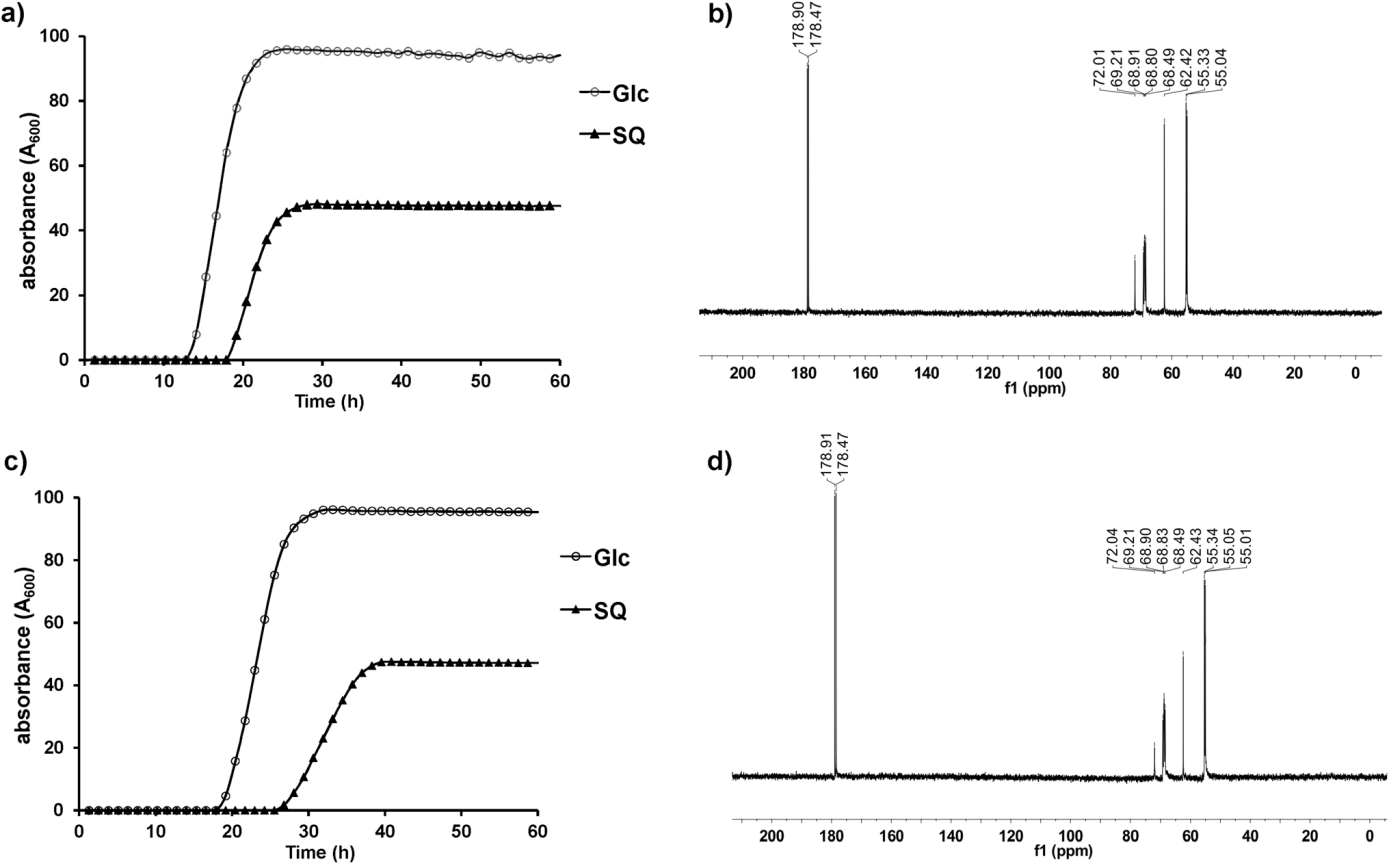
Growth curves of *Arthrobacter* strains **a** AK01 and **c** AK04 grown on minimal salts media containing 5 mM glucose or SQ. ^13^C-NMR (500 MHz) spectra of spent culture media of *Arthrobacter* strains **b** AK01 and **d** AK04 grown on ^13^C_6_-SQ (7.7 mM)

Genome analysis of *Arthrobacter* spp. AK01 and AK04 revealed a cluster of genes that were assigned as encoding SQ degradation through the sulfo-EMP pathway (Fig. 3a, b). The prototypical sulfo-EMP pathway was identified in *E. coli* and involves a 10-gene cluster (*yihOPQRSTUV*, *csqR*) (Denger et al. 2014). In *E. coli* these genes encode a transcription factor (CsqR) (Shimada et al. 2019), putative transmembrane proteins for the import of SQ and export of the end-product of the pathway, DHPS (YihO, YihP). The enzymatic steps involve a sulfoquinovosidase (YihQ) for cleavage of SQ glycosides (Speciale et al. 2016), sulfoquinovose mutarotase (YihR) for interconversion of SQ anomers (Abayakoon et al. 2018b), SQ-sulfofructose (SF) isomerase (YihS) that interconverts SQ, SF and sulforhamnose (Sharma et al. 2021), an ATP-dependent sulfofructose kinase (YihV) that converts SF to SF-1-phosphate (SFP) (Sharma et al. 2021), SFP aldolase (YihT) which converts SFP to SLA and dihydroxyacetone phosphate (Sharma et al. 2021), and an NADH-dependent SLA reductase (YihU) to convert SLA to DHPS (Sharma et al. 2020), which is excreted into the growth media (Fig. 3d).

Both *Arthrobacter* sp. contained genes encoding SQ-SF isomerase (YihS), SF kinase (YihV), and SFP aldolase (YihT). Consistent with the excretion of SL into the growth media, both strains lacked an SLA reductase homologue, but instead contained an SLA dehydrogenase homologue, annotated as GabD. GabD homologues within sulfoglycolytic gene clusters have been identified for bacteria that utilize the sulfo-ED (Felux et al. 2015; Li et al. 2020) and sulfo-SFT (Frommeyer et al. 2020; Liu et al. 2020) pathways, and in *Pseudomonas putida* SQ1 there is an NAD^+^/NADP^+^-dependent SLA dehydrogenase (Felux et al. 2015). Recently, a sulfo-EMP pathway in *Bacillus urumqiensis* was identified that contained a GabD homologue, SlaB, however, it was unable to be recombinantly expressed and its activity is therefore unknown (Liu et al. 2021); we note that SlaB from *Desulfovibrio* sp strain DF1 could be recombinantly expressed and was confirmed to be an NADH-dependent, SL-producing SLA dehydrogenase (Burrichter et al. 2018). A proposed pathway for SQ metabolism in these *Arthrobacter* spp. is shown in Fig. 3c. AK01 and AK04 represent the first characterized examples of sulfoglycolytic bacteria that use a sulfo-EMP pathway but with an SLA dehydrogenase instead of SLA reductase, as described for the prototypical sulfo-EMP pathway in *E. coli* (Denger et al. 2014). *E. coli* and related Enterobacteriacae that contain SLA reductases are facultative anaerobes, and the presence of a reducing pathway for excretion of DHPS may support their anaerobic lifestyle. On the other hand, *Arthrobacter* are normally considered aerobes (Jones and Keddie 2006) (although anaerobic *Arthrobacter* have been described (Eschbach et al. 2003)), which is consistent with an oxidative pathway for excretion of SL.

SQ import and DHPS/SL export is poorly characterized, but several different strategies have been identified across diverse sulfoglycolytic bacteria. Both AK01 and AK04 contain genes annotated as ABC transporter cassettes and solute binding proteins. ABC transporter systems have been identified in *Agrobacterium tumefaciens* C58 (which uses the sulfo-SMO pathway) (Sharma et al. 2022) and *R. leguminosarum* SRDI858 (which utilizes a sulfo-ED pathway) (Li et al. 2020). The *A. tumefaciens* solute binding protein binds SQGro with high affinity (Sharma et al. 2022). We, therefore, propose that these *Arthrobacter* isolates utilize the solute binding protein and an ABC transporter system to import SQ or its glycosides. Strain AK04 also contains a TauE permease (a member of the 4-toluene sulfonate uptake permease (TSUP) system) (Shlykov et al. 2012). TSUP proteins are poorly characterized permeases that are suggested to be involved with the transport of sulfur-containing organic compounds. TauE of *Cupriavidus necator* H16 is proposed to be involved in the export of sulfolactate (Weinitschke et al. 2007), a function that is consistent with the excretion of SL by AK04. The absence of an obvious permease candidate in the AK01 gene cluster suggests that another protein may adopt this function in this strain.

Other key differences with *E. coli* includes the lack of identifiable SQ mutarotase and SQase encoding genes. Interestingly, the isolated strains could grow on the simple SQ glycoside, methyl α-sulfoquinovoside. This suggests that they may harbor an unidentified SQase without homology to known SQases. Finally, both organisms contained a gene encoding an IolG homologue, of unknown function. IolG proteins are NAD(P)-dependent oxidoreductases of the Gfo/Idh/MocA family, and catalyze oxidation of the hydroxyl groups of pyranose and inositol rings (Taberman et al. 2016). Members of this family include inositol dehydrogenase (Idh), which forms 2-keto-myo-inositol (2-inosose) from *myo*-inositol (Ramaley et al. 1979; Yoshida et al. 2006), glucose-6-phosphate dehydrogenase (G6PD), which forms 6-phosphogluconolactone from glucose-6-phosphate (Rowland et al. 1994), and levoglucosan dehydrogenase, which forms 3-keto-levoglucosan from levoglucosan (1,6-anhydro-β-d-glucose) (Sugiura et al. 2018; Kuritani et al. 2020). Inositol dehydrogenase IolG from *Bacillus subtilis* has maximal activity on *myo*-inositol and possesses activity on d-glucose and d-xylose, and produces d-gluconolactone from the former (Ramaley et al. 1979). Possibly, the *Arthobacter* spp. IolG homologues may convert SQ to SGL, which coincidentally is an intermediate in the sulfo-ED pathway (Felux et al. 2015), although the downstream sulfo-ED genes are missing in these organisms. An alternative possibility is suggested by the Gfo/Idh/MocA family member DgpA from the intestinal bacterium PUE. DgpA catalyzes the oxidation of the 3-hydroxyl group of the C-glycoside puerarin, facilitating the elimination of the aglycon and formation of 3-keto-2-hydroxyglucal (1,5-anhydro-d-*erythro*-hex-1-en-3-ulose) (Nakamura et al. 2020). A similar process applied to an SQ glycoside could facilitate the cleavage of the glycoside in the absence of an SQase and would give rise to the corresponding 6-sulfo-3-keto-2-hydroxyglucal, the fate of which is uncertain. However, we were unable to identify possible companion genes encoding proteins that would be required to enable the elimination/isomerization/reduction to SQ.

**Fig. 4:**
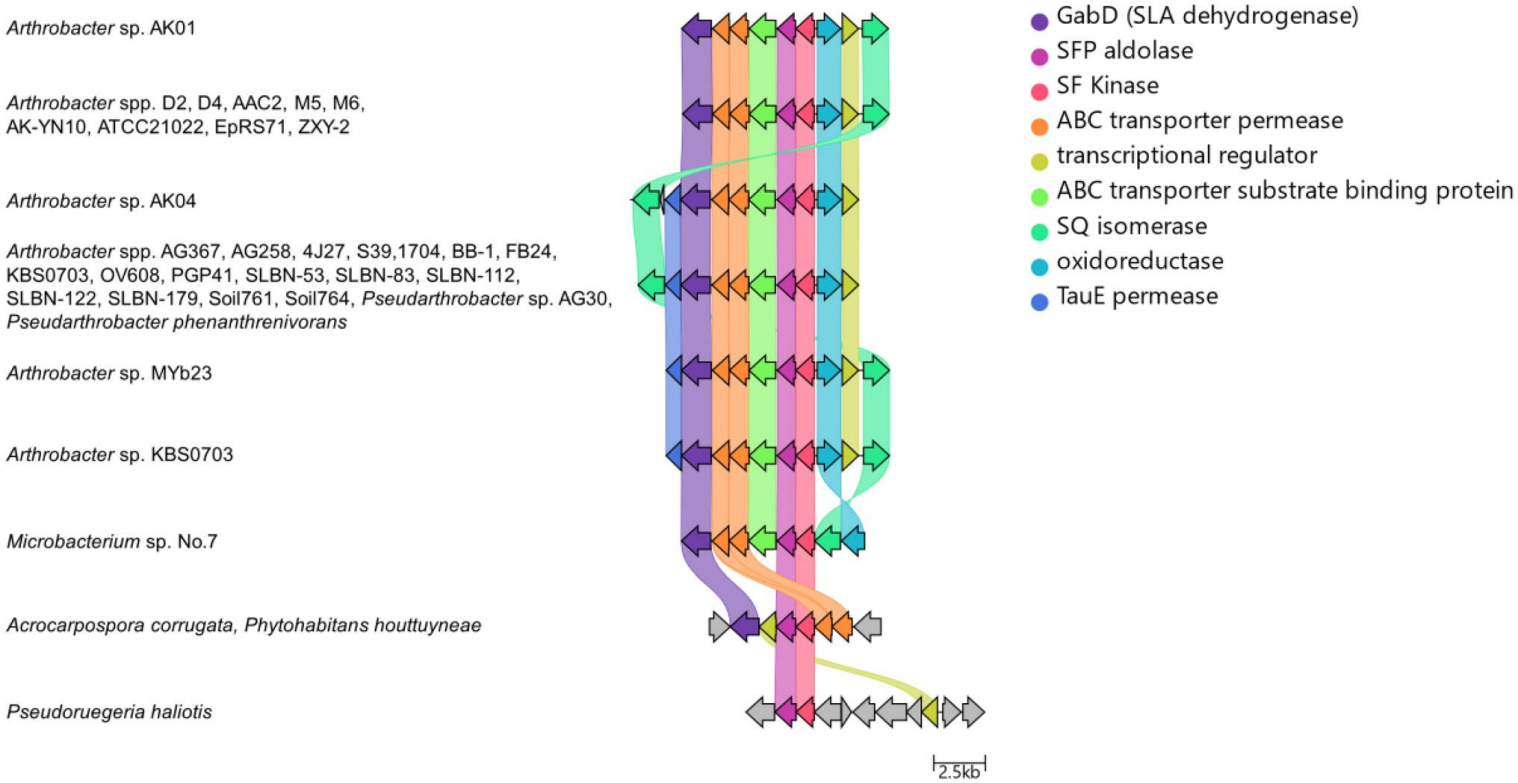
Distribution and architecture of sulfo-EMP gene clusters in *Arthrobacter* and related organisms. Syntenic relationship of sulfo-EMP gene clusters in *Arthrobacter* sp. AK01 and AK04 with homologous gene clusters. Colored links indicate ≥ 30% protein sequence similarity. Genome accession codes: *Arthrobacter* sp. D2 (LUKB01000109.1), *Arthrobacter* sp. D4 (LUKC01000078.1), *Arthrobacter* sp. AAC2 (JAAGBD010000014.1), *Arthrobacter* sp. M5 (LVCB01000107.1), *Arthrobacter* sp. M6 (LVCC01000103.1), *Arthrobacter* sp. AK-YN10 (AVPD02000157.1), *Arthrobacter* sp. ATCC 21,022 (CP014196.1) *Arthrobacter* sp. EpRS71 (LNUV01000003.1), *Arthrobacter* sp. ZXY-2 (CP017421.1), *Arthrobacter* sp. AG367 (VIVE01000010.1), *Arthrobacter* sp. AG258 (SOBI01000009.1), *Arthrobacter* sp. 4J27 (CAQI01000048.1), *Arthrobacter* sp. S39 (SIHX01000007.1), *Arthrobacter* sp.1704 (SOBD01000016.1), *Arthrobacter* sp. BB-1 (VDEV01000010.1), *Arthrobacter* sp. FB24 (CP000454.1), *Arthrobacter* sp. KBS0703 (MVDG02000001.1), *Arthrobacter* sp. OV608 (FOEZ01000003.1), *Arthrobacter* sp. PGP41 (CP026514.1), *Arthrobacter* sp. SLBN-53 (VFMZ01000001.1), *Arthrobacter* sp. SLBN-83 (VFMX01000001.1), *Arthrobacter* sp. SLBN-112 (VFMU01000001.1), *Arthrobacter* sp. SLBN-122 (VFMS01000001.1), *Arthrobacter* sp. SLBN-179 (VFNR01000001.1), *Arthrobacter* sp. Soil761 (LMSF01000007.1), *Arthrobacter* sp. Soil764 (LMSI01000008.1), *Pseudarthrobacter phenanthrenivorans* (CP002379.1), *Pseudarthrobacter phenanthrenivorans* (RBNH01000003.1), *Pseudarthrobacter phenanthrenivorans* (VHJD01000009.1), *Pseudarthrobacter* sp. AG30 (QEHL01000024.1), *Arthrobacter* sp. MYb23 (PCPR01000010.1), *Arthrobacter* sp. KBS0703 (MVDG02000001.1), *Microbacterium* sp. No. 7 (CP012697.1), *Acrocarpospora corrugata* (BLAD01000050.1), *Phytohabitans houttuyneae* (BLPF01000004.1), *Pseudoruegeria haliotis* (PVTD01000003.1)

A search for organisms with gene clusters related to strains AK01 and AK04 led to identification of other *Arthrobacter* strains with syntenic or closely related sulfo-EMP gene clusters (Fig. 4). Other *Arthrobacter* spp. were identified that contained gene clusters with architectures identical to AK01; and both *Arthrobacter* spp. and *Pseudarthrobacter* spp. were identified with gene clusters identical to AK04. Non-identical but closely syntenic sulfo-EMP gene clusters were observed in select Actinobacteria including other *Arthrobacter* spp. and *Microbacterium* sp. No. 7 (both order Micrococcales), and a more distantly related cluster in the actinobacteria *Acrocarpospora corrugata* (order Streptosporangiales) and *Phytohabitans houttuyneae* (order Micromonosporales).

## Conclusions

Two sulfoglycolytic soil bacteria belonging to the *Arthrobacter* genus (strains AK01 and AK04) were isolated from soil by enrichment culture involving growth on SQ. The stationary phase optical density of these bacteria when grown on SQ was approximately half that of growth on glucose, consistent with utilization of only three of the six carbons of SQ. Both possessed a variant of the sulfo-EMP pathway that uses an SLA dehydrogenase to produce SL that is secreted into the growth media, and which is proposed to arise from an SLA dehydrogenase (GabD). SL in turn becomes available for other members of the microbial community that specialize in its metabolism (Rein et al. 2005; Cook et al. 2006; Denger et al. 2009, 2012; Denger and Cook 2010). Prior to this work, the only sulfo-EMP pathway bacteria that have been characterized were a range of *E. coli* strains, which produce DHPS through the action of SLA reductase (Denger et al. 2014). Notably, strains AK01 and AK04 lack genes assigned as encoding an SQase, but nonetheless could grow on MeSQ, indicating the presence of a non-specific SQase, a novel SQase that is not readily identified by sequence homology, or a novel lyase pathway. This study highlights the core genes required for sulfoglycolysis (YihS, YihT, YihU, YihV) and constitutes the first examples of sulfo-EMP bacteria isolated from soil.

## Supplementary Information

Below is the link to the electronic supplementary material.

Supplementary file1 (DOCX 36 KB)

## Data Availability

The datasets generated and/or analyzed during the current study are available in the GenBank database with accession codes SAMN23041292 (strain AK01) and SAMN23041293 (strain AK04).

## Abbreviations

ABC: ATP-binding cassette
DHPS: 2,3-Dihydroxypropanesulfonate
NAD(P)H: Reduced nicotinamide adenine dinucleotide (phosphate)
SF: Sulfofructose
SL: Sulfolactate
SLA: Sulfolactaldehyde
SQ: 6-Deoxy-6-sulfo-d-glucose
SQase: Sulfoquinovosidase
SQDG: Sulfoquinovosyl diacylglycerol
SQGro: Sulfoquinovosyl glycerol
Sulfo-ED: Sulfoglycolytic Entner–Doudoroff
Sulfo-EMP: Sulfoglycolytic Embden–Meyerhof–Parnas
Sulfo-SFT: Sulfoglycolytic sulfofructose transaldolase
Sulfo-TK: Sulfoglycolytic transketolase
Sulfo-SMO: Sulfoglycolytic sulfoquinovose monooxygenase
TSUP: 4-Toluene sulfonate uptake permease

## Code availability

Scripts used for bioinformatic search pipeline are available on the GitHub repository, https://github.com/jmui-unimelb/Gene-Cluster-Search-Pipeline.
