# Supplementary material for: Genome sequences of Arthrobacter spp. that use a modified sulfoglycolytic Embden–Meyerhof–Parnas pathway
Source: Arch Microbiol. 2022 Feb 24;204(3):193. doi: 10.1007/s00203-022-02803-2 (PMC8873060; doi:10.1007/s00203-022-02803-2)
Supplement: Supplementary file 1 — Supplementary file1 (DOCX 36 KB) [file 203_2022_2803_MOESM1_ESM.docx]

**SUPPLEMENTARY INFORMATION**

**Genome sequences of *Arthrobacter* spp. that use a modified sulfoglycolytic Embden-Meyerhof-Parnas pathway**

Arashdeep Kaur,^1,2^ Phillip L. van der Peet,^1,2^ Janice W.-Y. Mui,^1,2^ Marion Herisse,^3^ Sacha Pidot,^3^ Spencer J. Williams*^1,2^

^1^ School of Chemistry, University of Melbourne, Parkville, Victoria 3010, Australia.

^2^ Bio21 Molecular Science and Biotechnology Institute, University of Melbourne, Parkville, Victoria 3010, Australia

^3^ Department of Microbiology and Immunology, University of Melbourne, at the Peter Doherty Institute for Infection and Immunity, Victoria, 3000, Australia

E-mail: sjwill@unimelb.edu.au

**Table S1. Classification and general feature of *Arthrobacter* spp. AK01 and AK04**

| **MIGS ID** | **Property** | **Term** | **Evidence code** |
| --- | --- | --- | --- |
|  | Classification | Domain *Bacteria* | TAS [1] |
|  |  | Phylum *Actinobacteria* | TAS [2] |
|  |  | Class *Actinobacteria* | TAS [3] |
|  |  | Order *Actinomycetales* | TAS [3-6] |
|  |  | Family *Micrococcaceae* | TAS [3-5, 7] |
|  |  | Genus *Arthrobacter* | TAS [5, 8] |
|  |  | Species *Arthrobacter* sp. | TAS [2] |
|  |  | Strain: AK01, AK04 | IDA |
|  | Gram Strain | Not measured |  |
|  | Cell Shape | Short rod-like | IDA |
|  | Motility | Not reported |  |
|  | Sporulation | Not reported |  |
|  | Optimum Temperature | Not tested, used 30℃ | IDA |
|  | pH range; Optimum | Not tested, used 7-8 | IDA |
|  | Carbon source | Yeast extract/tryptone, glucose, sulfoquinovose | IDA |
| MIGS-6 | Habitat | Soil | IDA |
| MIGS-22 | Oxygen requirement | Aerobic | IDA |
| MIGS-15 | Biotic relationship | Free living | IDA |
| MIGS-4 | Geographic location | Melbourne, VIC, Australia | IDA |
| MIGS-5 | Sample collection | March 11, 2021 | IDA |
| MIGS-4.1 | Latitude | -37.7965449 | IDA |
| MIGS-4.2 | Longitude | 144.9595151 | IDA |
| MIGS-4.4 | Altitude | Not reported |  |

*Evidence codes - IDA: Inferred from Direct Assay; TAS: Traceable Author Statement (i.e., a direct report exists in the literature)

**Table S2. Gene comparison of putative sulfo-proteins.**

| Annotation | Accession code of comparator | AK01 strain | | AK04 strain | |
| --- | --- | --- | --- | --- | --- |
|  |  | **Locus tag** | **% Identity** | **Locus tag** | **% Identity** |
| Sulfoquinovose isomerase | BAE77429.1 | AK01_00271 | 59.56 | AK04_03995 | 58.92 |
| Sulfofructose kinase | BAE77426.1 | AK01_00274 | 31.76 | AK04_03987 | 32.32 |
| Sulfofructosephosphate aldolase | BAE77428.1 | AK01_00275 | 49.66 | AK04_03988 | 50.00 |
| Succinate-semialdehyde dehydrogenase [NADP(+)] GabD | WP_016486307.1 | AK01_00279 | 49.57 | AK04_03992 | 36.74 |

* comparators BAE77429.1, BAE77426.1, BAE77428.1 are from *E. coli* and WP_01648630 is from *Pseudomonas putida* SQ1

**References**

[1] Woese CR, Kandler O, Wheelis ML: Towards a natural system of organisms: proposal for the domains Archaea, Bacteria, and Eucarya. Proc Natl Acad Sci USA 1990;87:4576.

[2] Ludwig W, Euzéby J, Schumann P, Busse H-J, Trujillo ME, Kämpfer P, Whitman WB: Road map of the phylum Actinobacteria. In: *Bergey’s Manual® of Systematic Bacteriology: Volume Five The Actinobacteria, Part A and B.* Edited by Goodfellow M, Kämpfer P, Busse H-J, Trujillo ME, Suzuki K-i, Ludwig W, Whitman WB. New York, NY: Springer New York; 2012: 1-28.

[3] Stackebrandt E, Rainey FA, Ward-Rainey NL: Proposal for a New Hierarchic Classification System, Actinobacteria classis nov. 1997;47:479-91.

[4] Zhi X-Y, Li W-J, Stackebrandt E: An update of the structure and 16S rRNA gene sequence-based definition of higher ranks of the class Actinobacteria, with the proposal of two new suborders and four new families and emended descriptions of the existing higher taxa. Int J Syst Evolut Microbiol 2009;59:589-608.

[5] Skerman VBD, McGOWAN V, SNEATH PHA: Approved Lists of Bacterial Names. Int J Syst Evolut Microbiol 1980;30:225-420.

[6] Buchanan RE: Studies in the nomenclature and classification of the bacteria II. The primary subdivisions of the Schizomycetes. J Bacteriol 1917;2:155-64.

[7] Pribram E: A contribuiton to the classification of the microorganisms. J Bacteriol 1929;18:361-94.

[8] Conn HJ, Dimmick I: Soil Bacteria Similar in Morphology to Mycobacterium and Corynebacterium. J Bacteriol 1947;54:291-303.
